# Induction of Innate and Adaptive Immune Response against Recombinant HBsAg Protein Entrapped in Docosahexaenoic Acid Nanovesicles through Biomarkers

**DOI:** 10.3390/vaccines11020457

**Published:** 2023-02-16

**Authors:** Mohammed Ali Bakkari, Sivakumar S. Moni, Abdulrahman Alshammari, Muhammad H. Sultan, Osama A. Madkhali, Yosif Almoshari, Mohammad Firoz Alam, Mohamed Eltaib Elmobark

**Affiliations:** 1 Department of Pharmaceutics, College of Pharmacy, Jazan University, Jazan 45142, Saudi Arabia; 2 Department of Pharmacology and Toxicology, College of Pharmacy, King Saud University, Riyadh 11451, Saudi Arabia; 3 Department of Pharmacology and Toxicology, College of Pharmacy, Jazan University, Jazan 45142, Saudi Arabia

**Keywords:** vaccine adjuvants, recombinant HBsAg, immune response, cytokine network

## Abstract

The present study focused on demonstrating the induction of humoral and cell-mediated immunity through the establishment of a cytokine network. We hypothesized the anti-inflammatory, pro-inflammatory, and IgE antibody levels after vaccination with lyophilized recombinant HBsAg-loaded docosahexaenoic acid nanovesicles (LRPDNV), and the efficacy compared well with standard commercial recombinant hepatitis B vaccine. The cytokine network was efficiently regulated by striking a balance between pro-inflammatory cytokines IL-6, IL-8R, and IL-12 and anti-inflammatory cytokines such as IL-2, IL-4, IL-10, and IFN-γ immune response on the 14^th^ and 30^th^ day after primary and booster immunization. The acute phase protein CRP level was increased due to IL-6 after immunizing with LRPDNV. On the other hand, the IgE level was not significantly increased to induce any allergic reactions after immunization with LRPDNV. The study concluded that after immunizing with LRPDNV, a significant immunological response was established, implying that DHA nanovesicles have significant potential as an adjuvant method for delivering recombinant HBsAg protein. On the other hand, following immunization with LRPDNV, the IgE level was not noticeably elevated enough to cause any adverse reactions. The study concludes that a robust immune response was developed after immunizing with LRPDNV and suggests that DHA nanovesicles have much potential to deliver recombinant HBsAg protein.

## 1. Introduction

Globally, hepatitis B virus infection is an acute as well as a chronic infectious disease leading to human morbidity and mortality and has been projected to cause approximately 820,000 deaths in 2019, mostly from cirrhosis and hepatocellular carcinoma [1,2,3]. An estimated 830,180 persons died from the disease worldwide in 2020 [4]. In 2040, 1.3 million individuals are projected to die from liver cancer, which is 56.4% more than in 2020 [5]. Recombinant proteins are increasingly being utilized in the biopharmaceutical industry. In the current era, scientists have been able to successfully synthesize many recombinant proteins that have the potential to either treat or prevent diseases [6]. Recombinant hepatitis B vaccine (HBsAg) is considered a better vaccine candidate than plasma-derived vaccine because of safety aspects. The presently available recombinant HBsAg has been proven for protection after childhood administration. However, there is no assurance that the three-dose schedule will give protection throughout life. A recent study reported that the spectrum of immunity is decreasing due to an increase in age, and adults who lose immune protection are still at risk of infection [2]. Despite the success of the universal vaccination program, the multiple dosage regimen is also facing non-responders for follow-up vaccination due to a lack of knowledge about the importance of booster doses, probably due to economic crisis or nonbelieving nature prevailing in social and cultural ethics across the world [7,8]. According to a recent study, non-response to immunization can be attributed to resistance caused by genes, old age, obesity, gender, chronic or/and systemic diseases, smoking habits, and medicines that weaken the immune system [8]. The adjuvants that are used in vaccine formulation are crucial to the idea that a vaccine can trigger the body’s natural immune response. During the last nine decades, many adjuvants have been developed to deliver antigens in their naïve form safely. Unfortunately, most of the adjuvants are not approved for human use either due to toxicity or inefficiency. Therefore, developing potent, safe adjuvant systems is focused on the single-shot vaccine as the most critical advancement in human health care. Therefore, the primary step in enhancing the effectiveness of recombinant vaccines is to select an ideal adjuvant. Our previous study suggested that docosahexaenoic acid nanovesicles exhibited excellent physicochemical characterization and were able to induce a good spectrum of specific immune responses and concluded that they are a better adjuvant for recombinant HBsAg protein [9]. The purpose of the current work was to elucidate the molecular basis of the immune response to DHA nanovesicles used to deliver recombinant HBsAg protein.

## 2. Materials and Methods

### 2.1. Materials

All kits used in this study were purchased from MyBiosource, San Diego, CA, USA, and Abcam, Cambridge, MA, USA. Ejadah Medical Supplies Est., Riyadh, Saudi Arabia, provided the kits and chemicals.

#### In Vivo Protocol

This is a continuation of our earlier research [9]. The protocol for the in vivo experimentation has already been described. Lyophilized recombinant HBsAg-loaded docosahexaenoic acid nanovesicles (LRPDNV) were created and used as the test vaccine, and the efficacy was compared with the standard marketed vaccine. The animals were divided into four groups of six rats each, with the following protocol:

Group 1: Normal group—the animals did not receive LRPDNV or vehicles.
Group 2: Product treatment group—the LRPDNV vaccine was administered intraperitoneally to the animals at a volume of 0.5 mL, which was equivalent to 1 mcg.
Group 3: Vehicle treatment group—the animals were immunized with 0.5 mL of lyophilized nanovesicles intraperitoneally.
Group 4: Standard vaccine treatment group—the animals were immunized with 0.5 mL of marketed HBsAg (1 mcg) vaccine intraperitoneally.

The immunogenicity analysis lasted for 30 days; on the 14^th^ day, the animals in Groups 2, 3, and 4 received a booster dosage using identical test samples. On the 14^th^ and 30^th^ days, blood samples were taken using a capillary tube from the retroorbital plexus. Before being analyzed, sera were separated with centrifugation and kept at 20 °C.

### 2.2. Determination of Biological Markers

#### 2.2.1. Interleukin-2 (IL-2)

The serum IL-2 concentration was quantified using a rat IL-2 ELISA kit, ABCAM, USA. This assay utilized a 96-well plate coated with an anti-IL-2 antibody. The immobilized antibody bound the IL-2 present in a sample to the appropriate wells after 100 μL of standards and samples were added into each well. At room temperature, the plate was covered and incubated for 2.5 h. Using a microplate strip washer, the plate was washed 4 times with 1× wash solution. The plate was inverted on clean paper towels to become dry, and in each well, 100 μL of 1× biotinylated IL-2 antibody was added and incubated for 1 h at room temperature. The reaction solution was discarded, and washing was repeated as described. A total of 100 μL of 1× HRP-Streptavidin solution was added to each well and incubated for 45 min at room temperature with gentle shaking. The reaction solution was discarded, and the washing procedure was repeated. In each well, 100 μL of 1× HRP-Streptavidin solution was added and incubated for 45 min at room temperature with gentle shaking. The wells were washed again as described and filled with 100 μL of the substrate (TMB) and placed on a plate agitator before being incubated at room temperature for 10 min in the dark. Finally, 50 μL of stopping solution was added to each well to terminate the reaction. The absorbance was measured at 450 nm using Biotek ELx800 (ELISA plate reader), Winooski, VT, USA, after vigorous shaking. The concentration of IL-2 was expressed in pg/mL, which was determined by extrapolating a standard curve.

#### 2.2.2. Interleukin-4 (IL-4)

A sandwich enzyme-linked immunosorbent assay was utilized to analyze the serum IL-4 levels quantitatively in vitro using a rat IL-4 kit, Abcam, USA. A total of 100 µL of standards and samples was pipetted into the wells, and IL-4 present in a sample was bound to the wells by the immobilized antibody. Similar to the methodology described for IL-2 determination, the IL-4 concentration was estimated by extrapolating a standard curve, and the concentration was expressed in pg/mL.

#### 2.2.3. Interleukin-10 (IL-10)

Simple stepwise procedures were used to achieve a quantitative measurement of the serum IL-10 levels using a rat IL-10 ELISA kit, Abcam, USA. In ELISA, the sample analyte is immunocaptured in solution using an affinity tag labeled capture antibody and a reporter conjugated detecting antibody. This complete combination is then immobilized using immunoaffinity, which is provided by an anti-tag antibody that coats the well. A total of 50 μL of standards and samples were added into each well, and 50 μL of antibody cocktail was added to each well. The plate was covered with a plate covering and shaken at 450 rpm for 1 h at room temperature. Using a microplate strip washer, the plate was washed 4 times with 1× wash solution 3 times. The wells were washed again as described and filled with 100 μL of the substrate (TMB) and placed on a plate agitator before being incubated at room temperature for 10 min in the dark. Finally, 100 μL of stopping solution was added to each well to terminate the reaction. The absorbance was measured at 450 nm using Biotek ELx800 (ELISA plate reader), USA, after vigorous shaking. The IL-10 concentration was calculated by extrapolating a standard curve, and the concentration was represented in pg/mL.

#### 2.2.4. Interferon Gamma (IFN-γ)

The in vitro Simple Step ELISA was used for determining the IFN-γ protein levels in the rat serum using a rat IFN-γ ELISA kit, ABCAM, USA. During the procedure, 50 µL of the standard or sample was injected into the corresponding well of the strip. Subsequently, 50 µL of the antibody mixture was added. The wells were sealed, and the plate was incubated (with agitation) for an hour. Then, a microplate strip washer was used to rinse it 3 times with 1× wash buffer (Biotek ELX50, USA). The wells were then filled with 100 μL of the substrate TMB and placed on a plate agitator before being incubated at room temperature for 10 min. A total of 100 μL of stopping reagent was used to stop the reaction process. An ELISA reader (Biotek ELx800, USA) was then utilized to obtain a reading of the optical density of each well at 450 nm, and the concentrations of IFN-γ were determined by extrapolating from a standard curve and expressed in pg/mL.

#### 2.2.5. Interleukin-6 (IL-6)

The level of L-6 was determined using an IL-6 ELISA kit, and a simple step-by-step ELISA test was established for the quantitative determination of IL-6 protein in rats. A total of 50 µL of standard and samples were placed in the appropriate wells. Then, 50 µL of antibody cocktail was added to each well. The plate was sealed and incubated for 1 h at room temperature by keeping it on a plate shaker set to 400 rpm. The plate was washed 3 times with 1× wash buffer PT using an ELISA washer, Biotek ELX50, USA. The plate was inverted on clean paper towels to become dry. The wells were then filled with 100 μL of the substrate TMB and placed on a plate agitator before being incubated at room temperature for 10 min in the dark. The reaction was stopped with 100 µL of stopping reagent. The optical density of each well was then read at 450 nm using Biotek ELx800 (ELISA reader, USA), and the concentration of IL-6 was determined by extrapolating from a standard curve and expressed in pg/mL.

#### 2.2.6. Interleukin 8 Receptor Alpha (IL-8 R)

The IL-8 R level was evaluated quantitatively using a rat IL-8 R ELISA kit, MyBiosource, USA. The kit utilizes a sandwich enzyme immunoassay for the in vitro quantitative detection of IL-8 R in rat serum. A total of 100 µL of standard and samples were placed in the respective wells of the strips. The plate was sealed and incubated at 37 °C for 90 min. After incubation, the liquid was removed from each well, 100 µL of detection A was added, and the plate was sealed with plate sealer. The plate was incubated at 37 °C for 45 min. The strips of the plate were washed thoroughly with 1× wash buffer 3 times using a microplate strip washer (Biotek ELX50, USA). After washing, 100 µL of detection B was added, and the plate was sealed with plate sealer. Then, the plate was incubated at 37 °C for 45 min. The plate was washed thoroughly with 1× wash buffer 5 times using a microplate strip washer (Biotek ELX50, USA). Each well was filled with 90 µL of substrate solution, and the plate was incubated at 37 °C for 20 min. Then, 50 µL of stopping solution was added to each well, and the plate was scanned at 450 nm using an ELISA reader (ELx800, USA). The concentration of IL-8R was calculated by extrapolating the standard curve, and it was expressed in pg/mL.

#### 2.2.7. Interleukin-12 (IL-12)

The level of IL-12 in serum was determined quantitatively using a rat IL-12 ELISA kit (Invitrogen Rat Interleukin-12 p40). It is a solid-phase sandwich enzyme-linked immunosorbent assay. A total of 100 µL of standard and samples were placed in the respective wells of the strips. Then, 50 µL of incubation buffer was aspirated to mix the contents. Then, the plate was sealed and incubated at room temperature for 2 h. After incubation, the liquid was removed from each well. The plate was washed 4 times with 1× wash buffer using an ELISA washer, Biotek ELX50, USA. The plate was inverted on clean paper towels to become dry. After that, 100 µL of Rt IL-12 biotin conjugate solution was added into each well and mixed well. The plate was sealed and incubated for 1 h at room temperature. After incubation, the plate was thoroughly aspirated and washed 4 times with a 1× wash buffer using an ELISA washer. Then, 100 µL 1× Streptavidin-HRP solution was added to each well. The plate was sealed and incubated for 30 min at room temperature. After incubation, 100 µL stabilized chromogen was added to each well and incubated for 30 min in the dark, and finally, 100 µL of stop solution was added. The plate was scanned at 450 nm using an ELISA reader (ELx800, USA). The concentration of IL-12 was calculated by extrapolating the standard curve, and it was expressed in pg/mL.

#### 2.2.8. IgE Antibody

Quantitative analysis of the serum IgE concentration was performed through simple step ELISA. The IgE that is present in the samples has a reaction with the anti-IgE antibodies that have been adsorbed to the surface of the polystyrene microtiter wells. A total of 100 µL of standard and samples were placed in the respective wells and incubated at room temperature for 60 min. The plate was washed 3 times with 1× wash buffer using Biotek ELX50, USA. A total of 100 μL of 1× enzyme-antibody conjugate to each well was added for 60 min. After incubation, the plate was washed as described earlier. After incubating the plates for 10 min in the dark with 100 µL of TMB development solution. Then, 100 µL of stop solution was added to the reaction mixture to terminate the enzymatic reaction, and the optical density of each well was read at 450 nm, ELISA reader (Biotek ELx800, USA). IgE concentration was determined by extrapolating from a standard curve, and it was expressed as ng/mL.

#### 2.2.9. C-Reactive Protein (CRP)

CRP concentrations in serum were determined using an automated clinical chemistry analyzer, Beckman Coulter, Brea, CA, USA. 

### 2.3. Statistical Analysis

GraphPad Prism 9 software, San Diego, CA, USA, was used to determine the statistical significance using one-way analysis of variance (ANOVA). The values are reported as mean standard deviation, with the levels of significance taken as *p* < 0.05, *p* < 0.01, and *p* < 0.001.

## 3. Results and Discussion

The immune response is a complicated chain of events that is intricately regulated, and the immune response is triggered by the introduction of an antigen into the body. The immune system has rapidly adapted to pathogen-associated molecular patterns (PAMPs) and antigen-associated molecular patterns (AAMPs) to develop slower, specifically tailored adaptive immune responses. Due to the relatively delayed nature of adaptive immunity, the quicker responding innate immune responses serve as an essential initial line of defense [10]. Adaptive immunity, on the other hand, involves the selection and clonal expansion of immune cells that harbor made-to-order somatically rearranged receptor genes (such as T and B cell receptors). These receptor genes recognize antigens from the pathogen, and as a result, adaptive immunity can provide specificity and long-lasting immunological memory [11]. The release of inflammatory cytokines and the activation of APCs, such as macrophages and dendritic cells, are just two of the many outcomes of innate immune responses. These nonclonal responses also shape the immune system in preparation for the later emergence of certain adaptive immunological responses [12,13]. Vaccine development prioritizes adjuvants, which boost antigen immunogenicity. Potent adjuvants enhance immune responses and accelerate the generation of robust immune responses, maintain them for a longer period, induce local immune responses, produce antibodies with increased avidity and neutralization capacity, elicit cytotoxic T lymphocytes (CTLs), increase the response rate in lower responders, and decrease the amount of vaccine required [14,15]. In the present study, LRPDNV was successfully augmented immune response through the induction of several cytokines. Table 1 depicts the anti-inflammatory and pro-inflammatory cytokines levels on the 14^th^ day and 30^th^ day. 

**Figure 1:**
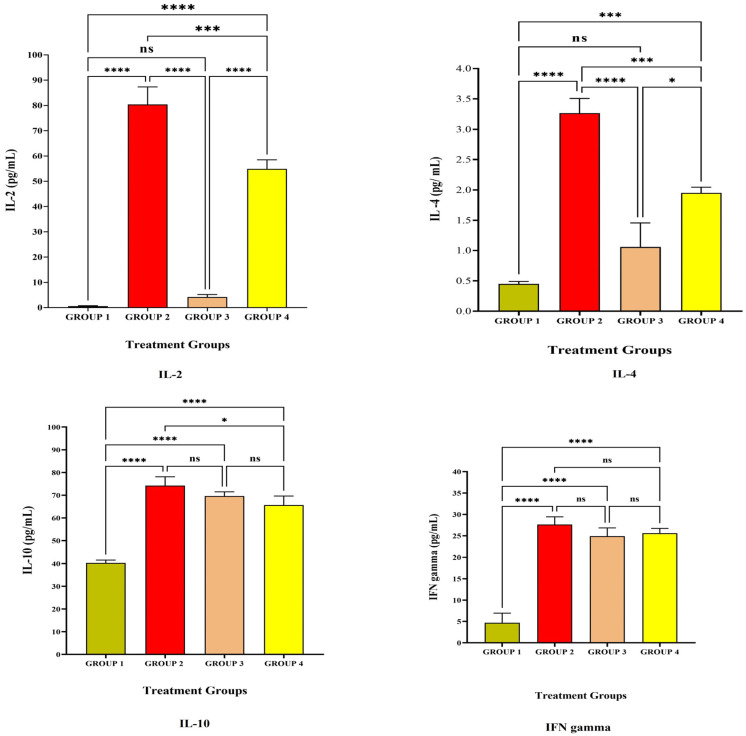
The study on anti-inflammatory cytokines against the immunization of LRPDNV on the 14^th^ day. **** Extremely high significant at *p* < 0.001, *** Highly significant at *p* < 0.05, * Significant at *p* < 0.05, non-significant (ns) at *p* < 0.05.

**Figure 2:**
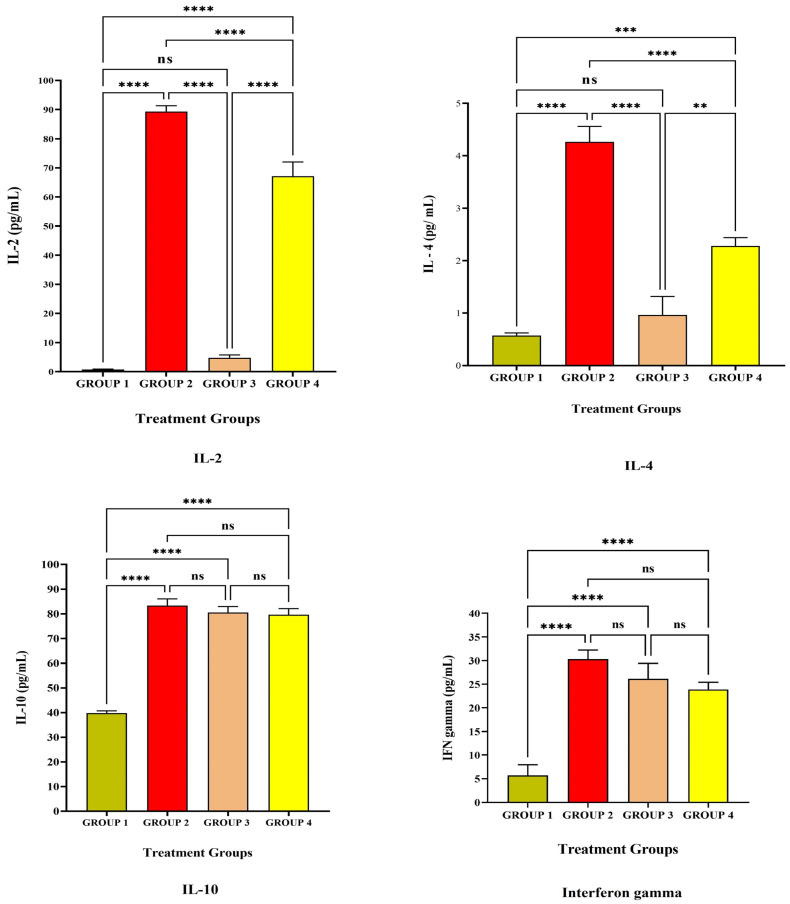
The study on anti-inflammatory cytokines against the immunization of LRPDNV on the 30^th^ day. **** Extremely high significant at *p* < 0.001, *** Highly significant at *p* < 0.05, ** Significant at *p* < 0.001, non-significant (ns) at *p* < 0.05.

Figure 1 and Figure 2 depict the anti-inflammatory cytokine study after LRPDNV immunization. Interleukin 2 (IL-2) is a lower molecular weight polypeptide that is produced by T cells during an immune response. It is essential for the development of effector T cells, including their expansion, proliferation, and differentiation from naive T cells. IL-2 is a protein that plays a pivotal role in the chain of events that comprise the immune response [16,17]. The anti-inflammatory cytokine study on the 14^th^ and 30^th^ days is depicted in Figure 1 and Figure 2, respectively. When compared to Group 1, the level of IL-2 in Group 2 was substantially very high at the *p* < 0.05 level on both the 14^th^ day and the 30^th^ day. On the other hand, at a threshold of *p* < 0.05, the level of IL-2 that was produced because of LRPDNV induction (Group 2) was significantly higher than the level produced by the commercially available standard vaccine (Group 4) on the 14^th^ day. On day 30, the levels of IL-2 were elevated in Group 2 and Group 4, respectively. On the other hand, the level of IL-2 was high due to LRPDNV induction (Group 2) when compared to the marketed standard vaccine (Group 4) at *p* < 0.05 level. The level of IL-2 was high in Groups 2 and 4 on the 30^th^ day after induction. However, the percentage increase of IL-2 in Group 2 on the 30^th^ day was 11.2% when compared to the 14^th^ day. In the standard vaccine group (Group 4), the % increase of IL-2 was 22.6 on the 30^th^ day when compared to the 14^th^ day. According to a previous study, HIV patients who received the normal dosage of the hepatitis B vaccine had higher levels of IL-2 induction than healthy controls. At 7 months after immunization, it was also shown that the healthy control group had an increased IL-2 level due to memory CD4+ T cells [18].

Interleukin-4 (IL-4) is a cytokine that is important for regulating anti-inflammatory reactions and growth factor synthesis in B and T cells [19]. The level of IL-4 was high in Group 2 when compared to Group 4 on both the 14^th^ and 30^th^ day after immunization with LRPDNV. When compared to Group 4, the level of IL-4 in treatment Group 2 was found to be significantly higher on days 14 and 30, achieving highly significant and very high significant levels at the *p* < 0.05 threshold of statistical significance. When compared to the 14^th^ day, the level of IL-4 in Group 2 had increased by 21.4% on the 30^th^ day. Conversely, the standard treatment group (Group 4) showed a 20% increase on the 30^th^ day compared with the IL-4 level on the 14^th^ day [20]. According to a previous study, IL-4 rs2243250 and IL-4 rs2227284 polymorphisms are associated with low anti-HBs Ab production following hepatitis B immunization in Korean newborns [21]. In our earlier investigation, both LRPDNV and the conventional vaccine elicited a good immune response, with LRPDNV exhibiting the highest anti-HBs level [9].

The anti-inflammatory cytokine interleukin 10 (IL-10) contributes significantly to the reduction of the host’s immune response to infections. On both the 14^th^ and 30^th^ day, all animal groups had a high level of IL-10. At a *p* < 0.05 significant threshold, Group 2 animals had significantly greater levels of IL-10 than Group 1. It is interesting to note that the level of IL-10 was found to be high when compared to Group 1, which indicated that the level of IL-10 was increased even in the absence of HBsAg. Moreover, the levels were found to be non-significant when compared to Group 2 and Group 4 at *p* < 0.05 level on the 14^th^ day. However, the IL-10 level was reduced on the 30^th^ day in Group 1 when compared to the rest of the groups. Following the booster dose, the percentage of patients in Groups 2, 3, and 4 who had elevated levels of IL-10 was 12.3, 15.6, and 21.8, correspondingly. This is an indicator that nanovesicles have been releasing HBsAg protein in a sustained manner. In an earlier investigation, it was found that the immune response to the HBV vaccine was substantially related to IL-10 gene variations. The identification of these high-risk infants, who were delivered to mothers who were positive for HBsAg but negative for HBeAg and had low responses to the hepatitis B vaccination [21]. According to the findings of another study, a particular variation in the IL-10 gene is connected to a chronic form of hepatitis B virus infection as well as liver damage. It is widely acknowledged that IL-10 plays a pivotal role in modulating the immunological response to HBV infection. Regulatory B-cells (B_reg_) are a subset of IL-10-producing B-cells that have recently been found to govern HBV-specific CD8+ Tcell immunity. Additionally, IL-10 downregulation restores the functionality of CD8+ T cells that are specific for HBV [22]. In contrast, our study demonstrated that the IL-10 level was considerable in Group 1, normal control, on both the 14^th^ and 30^th^ day. It is noteworthy that the level of IL-10 was down-regulating in Group 1, indicating the regulator B cells function in the regulation of immune response. However, IL-10 levels were not down-regulated in Groups 2, 3, and 4 on the 30^th^ day, probably due to the booster effect. An increased level of IL-10 was reported in people with a compromised immune system who retained anti-HBs after receiving the HBsAg immunization [23]. Similarly, in our earlier work, high levels of anti-HBsAg were seen in Groups 2 and 4 on the 30th day following vaccination [9].

Interferon-gamma (IFN-γ) is a glycosylated protein that is produced by Nk cells as well as helper T cells (CD4+) and cytotoxic cells (CD8 T+) cells [24]. IFN-γ is one of the most important immune and inflammatory mediators. It has a significant role in the activation of macrophages and the inflammatory process [25]. In the present study, The IFN-γ level was increased extremely significantly at *p* < 0.05 level when compared to Group 1. The levels of IFN-γ were non-significant at *p* < 0.05 level among Group 2, Group 3, and Group 4 on the 14^th^ day after vaccination. The level of IFN-γ was increased on the 30^th^ day after primary and secondary vaccination among Groups 2 and 3, but in Group 4, the standard vaccination group, a slight decrease in the level was observed. In response to a viral infection, the production of IFN-γ is an assurance of influencing both innate and adaptive immunity. In addition to playing a crucial part in the process of host defense, IFN-γ has also been linked to the etiology of chronic inflammatory disorders and autoimmune conditions when it is released in excessive amounts. IFN-γ, on the other hand, controls regulatory T and B cells, which have anti-inflammatory capabilities [26,27,28]. An earlier investigation found significant IFN- production against hepatitis B surface antigen when melittin was employed, and this was linked to the stimulation of both cell-mediated and humoral immune responses [29].

**Figure 3:**
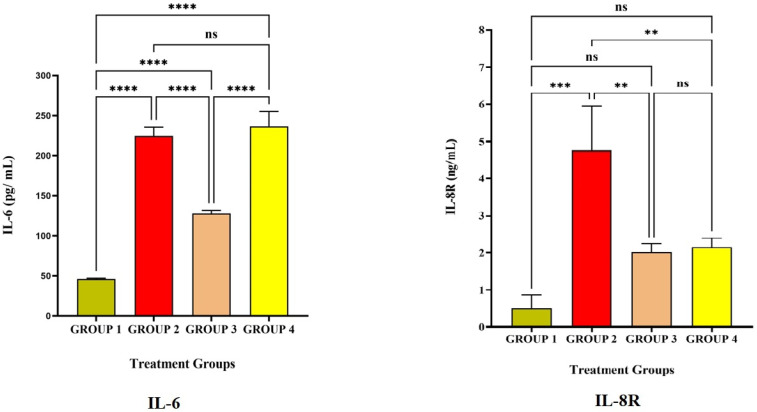
The study on pro-inflammatory cytokines against the immunization of LRPDNV on the 14^th^ day. **** Extremely high significant at *p* < 0.001 *** Highly significant at *p* < 0.05, ** Significant at *p* < 0.001, non-significant (ns) at *p* < 0.05.

**Figure 4:**
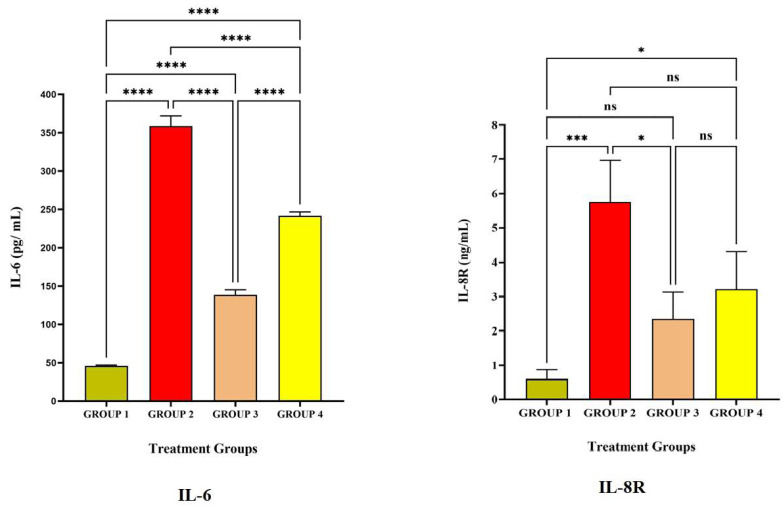
The study on pro-inflammatory cytokines against the immunization of LRPDNV on the 30^th^ day. **** Extremely high significant at *p* < 0.001 *** Highly significant at *p* < 0.05, * Significant at *p* < 0.05, non-significant (ns) at *p* < 0.05.

Figure 3 and Figure 4 represent the pro-inflammatory level after vaccination with LRPDNV on the 14^th^ and 30^th^ day. Interleukin-6 (IL-6) is a soluble mediator with a multifaceted influence on inflammation and immunological response [30]. In the present investigation, IL-6 levels in Group 2 were considerably greater than in Groups 1 and 3 (Table 1 and Figure 2). IL-6 levels did not differ significantly at *p* < 0.05 level between Groups 2 and 4. On day 14, however, the induction of IL-6 was 5.3% higher in Group 4 with the typical conventional vaccine compared to Group 2. It is interesting to note that, compared to the 14^th^ day, the level of IL-6 increased significantly by 59.5% on the 30^th^ day in Group 2. It is possible that this is because the booster dose caused a higher release of IL-6 from the macrophages. On the other hand, in Group 4, the increase in the amount of IL-6 that occurred after the booster dose was only 2% on the 30^th^ day compared to the 14^th^ day, which indicates that there was less induction of macrophages. An earlier study revealed that IL-6 might enhance the immunological response to hepatitis B immunization in newborns of HBsAg-positive mothers [31]. Another study indicated that hepatitis B vaccination is highly immunogenic and induces high levels of IL-6 and CRP in term newborns without sepsis who had their first vaccination shortly after birth [30]. The injection of LRPDNV increased the amount of IL-6 that was secreted by antigen-presenting cells macrophages, dendritic cells, neutrophils, B lymphocytes, and even some helper T cells (CD4+ Th_2_) cells during steady state, indicating the balance between innate and adaptive immunity [32,33].

IL-8 receptor (IL-8R) is generally expressed on neutrophils which is a pro-inflammatory progression [34,35,36]. The affinity of the receptor lies with IL-8, which is a component of innate immunity and is released by macrophages. The protein known as IL-8 is an essential component of the inflammatory response because of its critical function in the movement of neutrophils and other immune cells toward the site of an infection/antigen. In response to inflammation, neutrophils, macrophages, and monocytes all release IL-8, which is essential for activating neutrophils and attracting other immune cells to the infection site [37,38]. The higher level of IL-8R in Group 2 is extremely significant compared to Group 1 and significant compared to Group 4 at *p* < 0.05 level on the 14^th^ day and 30^th^ day. An earlier work indicated that IL-8 was most strongly up-regulated 8 weeks after HBV infection. The HBsAg protein activates IL-8 in the immune response to HBV [37]. Thus, the present study demonstrates the robust innate immune response through the induction of IL-8R.

**Figure 5:**
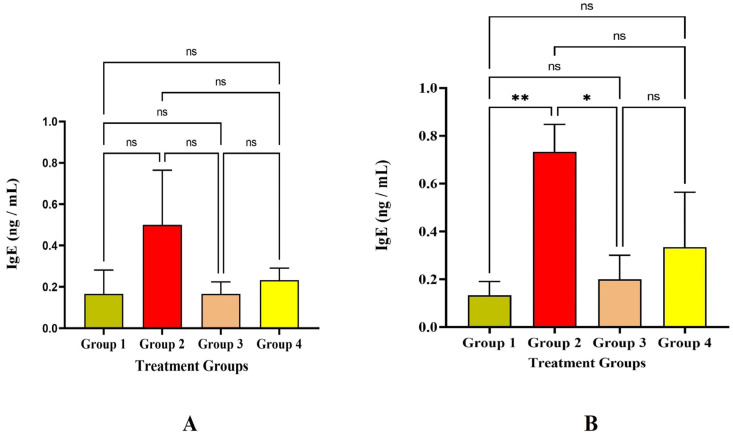
Study on IgE level (**A**) IgE level against the immunization of LRPDNV on the 14^th^ day (**B**) IgE level against the immunization of LRPDNV on the 30^th^ day. ** Significant at *p* < 0.001, * Significant at *p* < 0.05, non-significant (ns) at *p* < 0.05.

An antibody known as immunoglobulin E (IgE) is a marker for allergic reactions, and sensitization is the first step in the allergic immune response. In the present study, the IgE level among the four groups was significantly less, which was lesser than 0.5 ng/mL on the 14^th^ day, and IgE levels were increased on the 30^th^ day in Group 2 significantly at *p* < 0.05 level (Figure 5). CD4+ Th_2_ cells initiate the humoral immune response by stimulating antigen-specific B cells within the host to create IgE antibodies. Although there was a slight rise in IgE in Group 2, there was no major allergic reaction seen at the injection site, which was demonstrated by the histological analysis that we previously reported [9].

**Figure 6:**
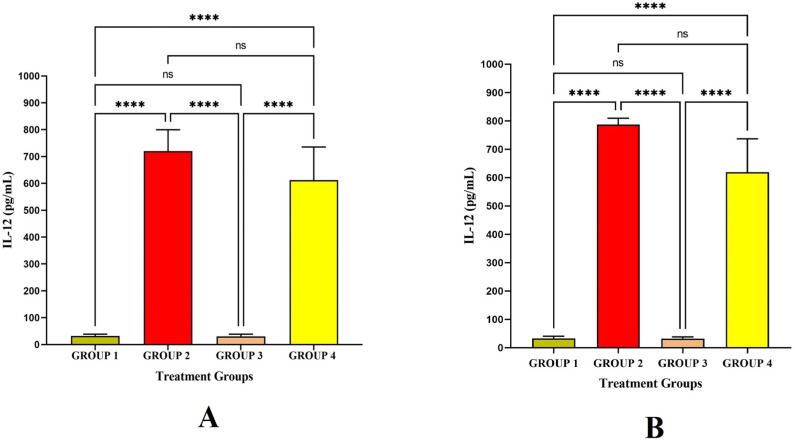
Study on Interleukin-12 (IL-12) Level. (**A**) IL-12 level against the immunization of LRPDNV on the 14^th^ day (**B**) IL-12 level against the immunization of LRPDNV on the 30^th^ day. **** Extremely high significant at *p* < 0.001, non-significant (ns) at *p* < 0.05.

Interleukin-12 (IL-12) is a crucial mediator between innate immunity and adaptive immunity and is a heterodimeric cytokine that controls T cell and natural killer-cell responses, triggers the generation of interferon (IFN), and favors the development of T helper _1_ (Th_1_) cells. In the present study, the IL-12 level was significant in Group 2 and Group 4 after LRPDNV injection on the 14^th^ and 30^th^ day. The level of IL-12 was non significantly higher in Group 2 when compared to Group 4 at *p* < 0.05 level (Figure 6). In contrast, the level of IL-12 was not increased significantly in Group 3 either on the 14^th^ or 30^th^ day after immunization of LRPDNV. An earlier study revealed that IL-12 was associated with an increased response to the hepatitis B vaccine in patients with chronic kidney disease who were on hemodialysis. This suggests that IL-12 can be utilized as a predictor of how well patients will respond to immunization [38]. According to a previous study, recombinant hepatitis B vaccination significantly increased IL-12 production among high responders compared to hypo-responders and non-responders. According to the findings of the study, there was a significant correlation between the levels of IL-12 and Th_1_ cytokines. This strongly suggests that IL-12 may play a substantial regulatory function in the control of Th_1_ cytokine production in high-responders [39]. Thus, the present study suggested that a robust IL-12 response was observed after LRPDNV immunization and maintained a high level even after 30 days of immunization.

**Figure 7:**
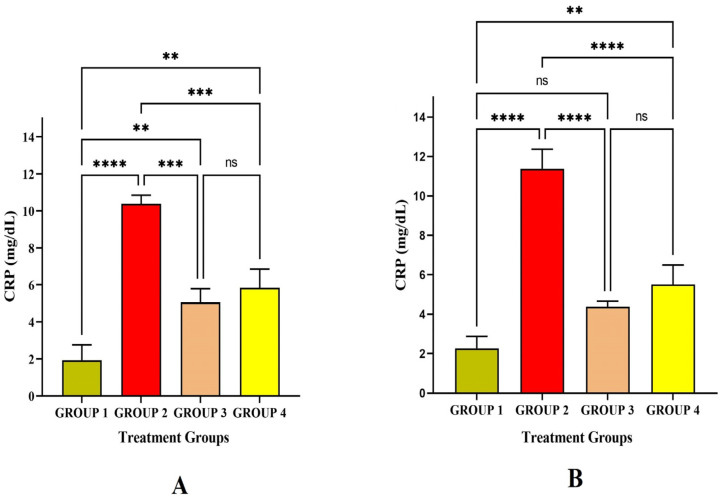
Study on CRP level (**A**) CRP level against the immunization of LRPDNV on the 14^th^ day (**B**) CRP level against the immunization of LRPDNV on the 30^th^ day. **** Extremely high significant at *p* < 0.001, *** Highly significant at *p* < 0.01, ** Significant at *p* < 0.05, non-significant (ns) at *p* < 0.05.

C-reactive protein (CRP) is an acute-phase protein produced by the liver due to the induction of inflammatory cytokines IL-6. In the present study, the CRP level significantly increased in Group 2 at *p* < 0.01 level on the 14^th^ and 30^th^ day after immunizing with LRPDNV when compared to the rest (Figure 7). Though both LRPDNV and standard vaccine induction showed a high level of IL-6 CRP, the level was increased very highly only in Group 2 due to LRPDNV induction. This confirms that LRPDNV formed a depot at the site of injection that causes some inflammatory reaction. However, in our previous study, the histopathological slides showed no adverse effect at the injection [9]. Therefore, induction CRP was due to the slow release of recombinant HBsAg from the site of injection. An early report indicated that hepatitis B vaccination causes CRP rise in term neonates following vaccination at birth [40]. In contrast, another study reported that hepatitis B vaccination did not affect the CRP level in healthy infants. In 2019, a study reported that the CRP level was elevated after vaccination with the HBsAg-adjuvanted vaccine HBsAg AS01B/Alum vaccine [41].

### Proposed Mechanism

**Figure 8:**
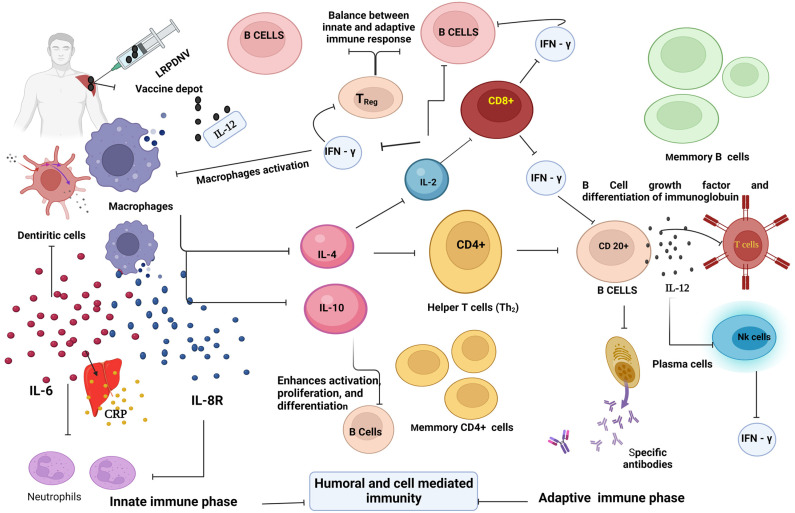
The mechanistic approach. This figure was created with BioRender.com, Bio Render, Canada.

Based on the outcome of our results, we propose a mechanistic approach to the development of immune response (Figure 8). The injectable formulation LRPDNV formed a depot at the site of injection. During innate immunity, the uptake of LRPDNV was initiated by macrophages and aggravated the releasing chemokine, IL-8R, produced by macrophages being activated. IL-8R is a potent neutrophil recruiting and activating factor. On the other hand, IL-6 is also secreted by dendritic cells in response to LRPDNV. IL-6 stimulates the neutrophils, which plays a key role in the development of innate immune responses. C-reactive protein (CRP) is a non-specific acute-phase protein that is generated in the liver by IL-6 induction due to antigenic stimulus. CRP plays a key role in the innate immune system by recognizing antigen-associated molecular patterns. CRP connects the innate and adaptive immune systems by activating complement and interacting with Fc gamma receptors. The activation of macrophages is also involved in the release of various cytokines during the innate phase of immune development. IL-4 and IL-10 are released by activated macrophages which regulate innate and adaptive immunity. The released IL-4 stimulates CD4+ (Th_2_) cells and B cells. The differentiation and stability of CD4+ cells are promoted by IL-4, while the differentiation of Th_1_ cells is inhibited. IL-4 is responsible for stimulating enhanced expression in resting B cells as well as an increase in IgG1 level. The production of an IgE response in vivo requires the participation of IL-4 as a crucial factor. IL-10 is involved in the activation and proliferation of B cells based on AAMPs. The activated CD4+ cells release IL-2 and increase the proliferation of activated B cells through surface receptors that are similar to those of activated T cells. Both IL-4 and IL-2 are involved in the regulation of immune response by recruiting CD8+ (Tc) cells. Furthermore, the integration of IL-2 and IL-4 signals helps to coordinate the different responses of regulatory T cells. Increased CD4+ T cell responses and immunogenicity in response to regulatory T cell depletion. Due to the activation of CD4+ and CD8+ cells during the adaptive phase, the IFN-γ was released.

Dendritic cells are part of a diverse family of immune cells that serve as a bridge between innate and adaptive immune responses. Dendritic cells are especially potent antigen-presenting cells that acquire antigens/microbial products during the innate immune phase and trigger adaptive immune responses against AAMP through cytokine induction. The presence of IL-4 during the process of dendritic cell formation, as well as the possession of IL-4 receptors on dendritic cells, can produce IL-4. On the other hand, IL-2 stimulates dendritic cell formation through innate and adaptive lymphoid cells. Dendritic cell maturation is essential for effective antigen presentation and the commencement of an immune response. Interleukin-12, often known as IL-12, is a heterodimeric pro-inflammatory cytokine that favors the development of T helper _1_ (Th_1_) cells, stimulates the generation of interferon-γ (IFN-γ), and acts as a link between innate and adaptive immunity. In response to antigenic stimulation, dendritic cells, macrophages, and B lymphocytes generate IL-12. IL-12 stimulates the proliferation of T and NK cells, as well as the augmentation of cell-mediated cytotoxicity and the production of cytokines, particularly IFN-γ. IL-12 and IL-12-induced IFN-γ are thought to be important in early immune responses. IL-12 is regarded as a mediator between innate and adaptive immunity since it increases IFN-γ production and aids in polarizing naive CD4+ T cells to become Th_1_ cells. In addition, IL-12 has been found to improve CD8+ T cell homeostasis, which increases the activation and survival of active CD8+ T cells.

IFN-γ is a macrophage-activating factor that activates macrophages and enhances the innate immune phase. Its immunomodulatory effects can be seen through improving leukocyte trafficking, presenting antigens more effectively, and digesting antigens through macrophages. IFN-γ is an essential Th_1_ cytokine, and the receptor of IFN-γ in dendritic cells signaling and suppresses the spontaneous proliferation of CD4+ T Cells. Multiple biological responses are coordinated by the IFN-γ signaling system, which is primarily involved in host defense and immunological surveillance but also in the development of adaptive immunity. IFN-γ is responsible for a diverse array of effects on the responses of mature B cells. These effects might be positive or negative. It not only functions as a B cell growth factor, but it also stimulates final differentiation, which leads to IgG production. It is crucial to comprehend how cytokines influence the growth of innate and adaptive immunity. As a result, cytokine measurements may potentially be used to forecast how innate and adaptive immunity will be regulated.

## 4. Conclusions

Docosahexaenoic acid (DHA) nanovesicles were created in this study as a recombinant HBsAg adjuvant system. Based on the findings of this molecular network, it appears that DHA nanovesicles have a promising future as a feasible adjuvant for recombinant HBsAg. Furthermore, when compared to the hepatitis B vaccine that is already on the market, the DHA nano vesicular system was found to be a superior adjuvant for the hepatitis B vaccination because it elicited an immune response that was strong and well-balanced on both the humoral and cell-mediated immune response through cytokine network. Although the molecular networking was determined in the current study through the cytokine network, it is necessary to access the receptor level studies as well as the cell signaling that takes place between CD4+, CD8+, CD20+, and T_reg_ cells to learn more about immune response, which will be the focus of the next level of research. Based on the outcome of current research, the docosahexaenoic acid nanovesicles adjuvant system has the potential to be utilized for a variety of antigens, specifically COVID-19 vaccines, to elicit a powerful and robust innate and adaptive immune response.

## Figures and Tables

**Table 1:** The biomarkers level.

Type of Cytokines	Group 1		Group 2		Group 3		Group 4	
	14^th^ Day	30^th^ Day	14^th^ Day	30^th^ Day	14^th^ Day	30^th^ Day	14^th^ Day	30^th^ Day
IL-2	0.6 ± 0.1	0.7 ± 0.15	80.4 ± 6.9	89.4 ± 1.9	4.17 ± 0.9	4.7 ± 0.9	54.8 ± 0.8	67.2 ± 4.8
IL-4	0.4 ± 0.04	0.6 ± 0.04	3.3 ± 0.2	4.2 ± 0.2	1 ± 0.3	0.9 ± 0.4	1.9 ± 0.09	2.28 ± 0.16
IL-10	40.3 ± 1.3	39.8 ± 0.9	74.2 ± 3.9	83.4 ± 2.7	69.6 ± 1.8	80.5 ± 2.4	65.6 ± 3.9	79.6 ± 2.4
IL-6	46.03 ± 0.3	45.9 ± 0.9	224.7 ± 10.7	358.6 ± 13.3	127.7 ± 3.8	138.7 ± 6.6	236.7 ± 18.4	241.62 ± 5.1
IL-8 R	0.5 ± 0.3	0.6 ± 0.2	4.76 ± 1.2	5.72 ± 1.1	1.96 ± 0.2	2.29 ± 0.8	2.15 ± 0.2	3.23 ± 1
IFN-γ	4.7 ± 2.2	5.7 ± 2.3	27.6 ± 1.8	30.3 ± 1.9	24.8 ± 1.8	26.1 ± 3.2	25.6 ± 1.1	23.9 ± 1.4
IL-12	33.3 ± 6.8	32.8 ± 7.8	746.2 ± 79.5	787 ± 22.3	28.9 ± 8.5	32.3 ± 5.7	659.4 ± 123.3	619.2 ± 117.6
CRP	2.3 ± 0.8	2.2 ± 0.6	10.3 ± 0.5	10.81 ± 0.9	5.45 ± 0.7	4.45 ± 0.2	6.4 ± 1	5.9 ± 0.9

Each value is the mean of six batches (n = 6) with standard deviation.

## Data Availability

The data used to support the findings of this study are included within the article.
